# A Polarity-Sensitive Far-Red Fluorescent Probe for Glucose Sensing through Skin

**DOI:** 10.3390/bios13080788

**Published:** 2023-08-04

**Authors:** Lydia Colvin, Dandan Tu, Darin Dunlap, Alberto Rios, Gerard Coté

**Affiliations:** 1 Department of Biomedical Engineering, Texas A&M University, College Station, TX 77843, USA; 2 Center for Remote Health Technologies and Systems, Texas A&M Engineering Experiment Station, College Station, TX 77843, USA

**Keywords:** polarity, competitive binding, glucose sensor, NIR, far-red, fluorescence, Concanavalin A, Cyanine 5.5, solvatochromism

## Abstract

The field of glucose biosensors for diabetes management has been of great interest over the past 60 years. Continuous glucose monitoring (CGM) is important to continuously track the glucose level to provide better management of the disease. Concanavalin A (ConA) can reversibly bind to glucose and mannose molecules and form a glucose biosensor via competitive binding. Here, we developed a glucose biosensor using ConA and a fluorescent probe, which generated a fluorescent intensity change based on solvatochromism, the reversible change in the emission spectrum dependent on the polarity of the solvent. The direction in which the wavelength shifts as the solvent polarity increases can be defined as positive (red-shift), negative (blue-shift), or a combination of the two, referred to as reverse. To translate this biosensor to a subcutaneously implanted format, Cyanine 5.5 (Cy5.5)-labeled small mannose molecules were used, which allows for the far-red excitation wavelength range to increase the skin penetration depth of the light source and returned emission. Three Cy5.5-labeled small mannose molecules were synthesized and compared when used as the competing ligand in the competitive binding biosensor. We explored the polarity-sensitive nature of the competing ligands and examined the biosensor’s glucose response. Cy5.5-mannotetraose performed best as a biosensor, allowing for the detection of glucose from 25 to 400 mg/dL. Thus, this assay is responsive to glucose within the physiologic range when its concentration is increased to levels needed for an implantable design. The biosensor response is not statistically different when placed under different skin pigmentations when comparing the percent increase in fluorescence intensity. This shows the ability of the biosensor to produce a repeatable signal across the physiologic range for subcutaneous glucose monitoring under various skin tones.

## 1. Introduction

Continuous digital monitoring of our health has been made possible through innovative advances within engineering, wearable technology, chemistry, materials science, computer science, and more [1]. Glucose monitoring is important for management of diabetes, a disease that impacts a large population. The prevalence of diabetes stands at an estimated 10.5% of the global population, or 536.6 million adults in 2021, as reported by the International Diabetes Federation (IDF), and by 2045, projections have estimated the number of adults living with diabetes to rise to 783.2 million [2]. Many health complications relating to blood vessels, nerves, eyes, kidneys, and the heart occur with glucose levels consistently outside of the normal range of 70 to 100 mg/dL (while fasting), leading to poor quality of life for a significant population size [3].

The monitoring of glucose levels allows for improved health outcomes. The commonly used finger-pricking method for blood glucose concentration tracking has known disadvantages of being painful and leaving individuals unaware of dangerous highs and lows that have occurred between measurements [4]. These concerning blood glucose levels can be tracked with a CGM using measurements taken automatically every 1–5 min and alarms are integrated to warn of hyper- or hypoglycemia [5]. Millions have adopted the use of CGM systems globally, with Abbott alone reporting upwards of 3.5 million users with their FreeStyle Libre technology [6]. DexCom and Medtronic join Abbott in the field of indwelling electrochemical-based sensors for CGM while Senseonics stands alone with their fully implantable optical sensing design [7]. Each of these CGMs rely on contact with the subcutaneous tissue and measure glucose in the interstitial fluid (ISF). The advantages of current market CGMs are great, but limitations exist on current sensors such as the need for daily calibration, uncomfortably large probe/implant size, adhesive adherence and comfort, and sensor replacements every 3 to 14 days for the indwelling electrochemical CGM devices [8]. The Senseonics Eversense is fully implantable and thus has a longer lifetime and avoids the issues with the adhesive but is still rather large since it includes both the assay and detection optoelectronics, thus requiring implantation rather than injection. In order to advance current CGM technologies, the sensor lifespan, miniaturization, sensitivity, reliability, and specificity must be enhanced [5].

These CGMs all act as biosensors consisting of three key elements: (1) recognition, (2) transduction, and (3) data evaluation. Bioreceptors such as enzymes, antibodies, cells, or nucleic acid can serve as the recognition element for glucose. The transduction element converts recognition events into a detectable, readable signal through electrochemical, optical, thermometric, magnetic, or piezoelectric means which is then analyzed and displayed via the data evaluation element [9].

Fluorescence has been heavily researched as a method for continuous glucose monitoring because of its high sensitivity, high selectivity, minimal invasiveness, long lifespan, reliability, and traceability [10]. For a subcutaneously fully injectable fluorescence biosensor with the light source and detector located outside of the body, the light’s interaction with skin such as absorbance, scattering, and reflectance must be taken into consideration [11]. Light absorption and scattering based on tissue morphology, melanin and subcutaneous lipids, lead to reduced light transmission through skin. Although red and NIR light show improved tissue depth penetration and decreased melanin absorption when compared to lower wavelengths, reduced transmittance has still been observed for both darker and thicker skin samples and must be further investigated in terms of an implantable biosensor [12]. Fluorescent probes within the far-red and NIR range can be designed to provide intensity-based detection by leveraging their response to their microenvironment such as the polarity, acidity/basicity, or fluidity [13,14]. The change in a molecule’s absorption and/or emission due to dissolution in solvents of varying polarity is called solvatochromism and has been researched widely in the field of fluorescent probes [13,15,16,17,18,19].

For fluorescence-based glucose sensing, there have been three consistently researched types of recognition molecules which include enzymes, boronic acid derivatives, and glucose-binding proteins [20]. ConA is a glucose-binding protein derived from the jack bean (Canavalia ensiformis). ConA is of value to continuous glucose biosensing because it can reversibly bind to mannose and glucose molecules without altering the protein’s covalent structures [21]. In addition, ConA has a high specificity to glucose and a ConA-based assay has demonstrated good specificity to glucose in the presence of galactose, fructose, lactose, and sorbitol [22,23,24,25]. Toxicology studies of ConA have previously been analyzed for the application of a minimally invasive glucose biosensor where the protein would be housed in a semipermeable membrane and showed an extremely low risk within the concentrations of interest [26]. One limitation of ConA is that it is prone to aggregate due to thermal denaturation or its multiple binding sites. Our group has successfully implemented two methods to reduce aggregation: (1) use a small molecule competing ligand (mannotetraose) which can only interact with one binding site on ConA at a time to prevent a lattice-type structure from forming, and (2) PEGylation of ConA to prevent unfolded ConA from aggregating to itself while trying to enclose the exposed hydrophobic residues of the protein [25,27]. Use of a small competing ligand and PEGylated ConA within a fluorescence anisotropy and fluorescence resonance energy transfer (FRET)-based assay showed improved sensitivity when compared to much larger dextran molecules and stability of the assay was recorded after 30-day incubation at 37 °C [28]. In addition to the mentioned work, there are other ConA-based assays using FRET [29,30,31]. However, FRET sensors rely on specific pairs of donor and receptor dyes that add more complexity to the sensor.

In this paper, we developed a glucose sensor using ConA and a far-red fluorescent probe, which generated a fluorescent intensity change based on solvatochromism. Polarity-based sensing mechanisms for glucose have been studied using various fluorescence dyes such as 651-Blue Oxazine, Nile Red, Rhodamine B, R-dye, and Auramine O [32,33]. We used a previously developed method to synthesize Cy5.5-labeled small mannose molecules [34]. These dye–sugar conjugates were designed as an improved alternative to the 8-aminopyrene-1,3,6-trisulfonic acid (APTS)-labeled mannotetraose. The APTS is excitable in the blue range (450 nm), leading to poor light penetration of the tissue. Cyanine 5.5 was chosen because of its excitation in the red range (680 nm), allowing for deeper light penetration and because it has specifically been utilized in various applications where in vivo mouse studies have shown low concerns of toxicity [35,36,37,38]. This is beneficial for our goal of a subcutaneous biosensor which utilizes fluorescence intensity as a means to monitor glucose concentration.

**Figure 1:**
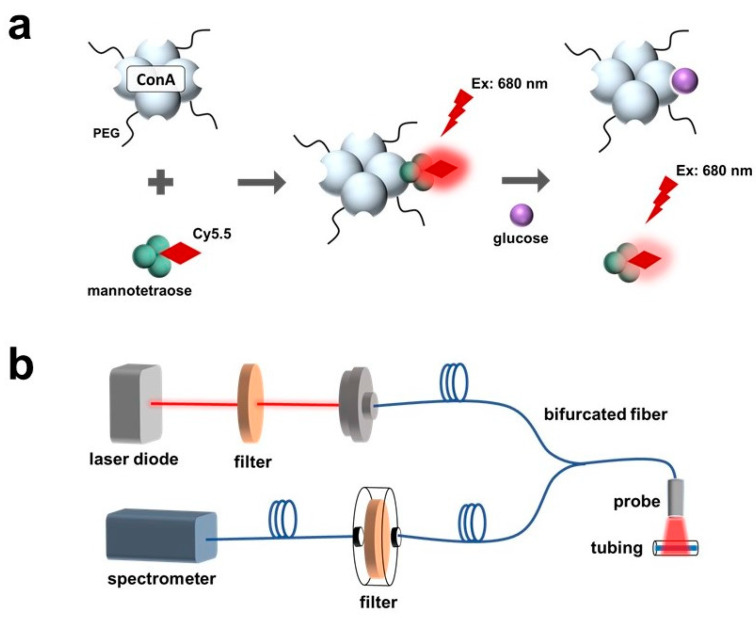
Schematic of a fluorescence-intensity-based glucose-sensing assay composed of PEGylated ConA and Cy5.5-labeled mannotetraose (**a**). Layout of optical benchtop system containing a laser diode, 700 nm shortpass excitation filter, bifurcated optical fiber, 700 nm longpass emission filter, and spectrometer for measurement of the assay within plastic tubing at low volumes of 2.5 μL (**b**).

In this work, the sensitive response of the Cy5.5-labeled mannose to polarity was observed and characterized. The polarity-sensitive Cy5.5-labeled mannose was then paired with PEG-ConA for glucose sensing (Figure 1a). The Cy5.5-labeled mannose functioned as a competing ligand to bind with PEG-ConA. Binding and unbinding of the Cy5.5-labeled mannose with PEG-ConA led to a fluorescent intensity change due to solvatochromism.

The developed sensor was tested with an optical benchtop system to measure the fluorescence intensity (Figure 1b). The performance of the fluorescent probe and the sensor when placed beneath samples of rat skin of varying thicknesses and pigmentations were investigated and compared.

## 2. Materials and Methods

### 2.1. Materials and Instrumentation

Concanavalin A, methyl-α-mannopyranoside (MaM), Methoxypolyethylene glycol 5000 propionic acid N-succinimidyl ester (mPEG-SPA), Tris-buffered saline (TBS) tablets, Amicon Ultra-2 Centrifugal Filter Units 30 kDa MWCO, sodium carbonate, sodium bicarbonate, dimethyl sulfoxide (DMSO), methyl alcohol (MeOH), anhydrous dextrose, and phosphate buffered saline (PBS) tablets were purchased from Sigma (St. Louis, MO, USA). Cyanine5.5 NHS ester (Cy5.5) was purchased from Lumiprobe (Hunt Valley, MD, USA). The sugar α1-3,α1-6 mannotetraose was purchased from Biosynth (Compton, UK), and α1-3,α1-6 mannotriose and α1-4 mannobiose were obtained from DextraUK (Reading, UK).

For the optical benchtop setup, the Ocean Optics USB4000 Spectrometer and 400 µm Reflection Probe were purchased from Ocean Optics (Dunedin, FL, USA). The NI myDAQ was obtained from National Instruments (Austin, TX, USA). A red 690 nm laser diode, adjustable fiber optic stand, filter holder, 700 nm shortpass and longpass filters, and miscellaneous benchtop accessories were purchased from Thorlabs, Inc. (Newton, NJ, USA). The power supply, SparkFun MOSFET Power Control Kit, and SparkFun Breadboard Power Supply Stick 5 V/3.3 V were purchased from SparkFun (Niwot, CO, USA). All tissue samples were obtained through the Tissue Share Program at Texas A&M University.

### 2.2. Synthesis of Cy5.5-Mannose and PEG-ConA

The synthesis protocol for the Cy5.5-mannose conjugates and PEGylated ConA has been described in detail in previous work [25,34]. The Cy5.5-labeled mannose molecules were synthesized using a linker molecule and a three-step procedure. PEGylation of ConA was conducted through utilization of the primary amines on ConA. The synthesis steps are detailed in the Supporting Information.

### 2.3. Binding Studies of PEG-ConA and Cy5.5-Mannose

To demonstrate the change in fluorescence intensity that occurs when PEG-ConA is added to Cy5.5-mannotetraose, Cy5.5-mannotriose, and Cy5.5-mannobiose, serial dilutions of PEG-ConA were added to a steady concentration of the Cy5.5-labeled mannose. Fluorescence emission data were collected using an Infinite 200 Pro plate reader (Tecan, Männedorf, Switzerland) and 96-well, flat bottom, black Costar well plates (Corning). For each run, the excitation wavelength was set to 655 nm instead of the optimal 680 nm due to the light source bleed-through present with the instrument; however, due to the broad nature of the absorbance peak of Cy5.5, a detectable emission spectrum was observed. An emission wavelength range of 698 to 714 nm was used to determine the peak emission values used in all calculations. The percent change in fluorescence intensity was calculated by comparing the peak emission of Cy5.5-labeled mannose in TBS to that of the peak emission with PEG-ConA present. The methods for this are described in the Supporting Information.

A red-shift of the fluorescence emission of each Cy5.5-mannose complex was observed as increasing concentrations of PEG-ConA were introduced. To demonstrate this red-shift, emission spectra (680 nm–800 nm) were collected for 100 nM Cy5.5-mannotetraose (λex: 655 nm) with concentrations of 0, 0.016, 0.031, 0.063, and 4.00 μM. This red-shift was further analyzed with Cy5.5-mannobiose, Cy5.5-mannotriose, and additional concentrations of PEG-ConA using identical plate reader settings.

The impact on fluorescence emission intensity of 100 nM Cy5.5-mannotetraose with the addition of unmodified ConA was studied using similar methods detailed above with PEGylated ConA. The ConA solution was prepared by dissolving 10 mg of ConA into 1 mL of TBS and mixing slowly until dissolved fully. The concentration was confirmed prior to preparing the 11 serial dilutions beginning at 24 μM and all measurements were collected within 4 h of initially dissolving the ConA to reduce instances of aggregation. All plate reader settings and experimental methods were identical to the previously described PEG-ConA binding studies, and the percent change in the peak fluorescence emission intensities was calculated.

### 2.4. Characterization of the Effect of Solvents on the Cy5.5-Mannose

To determine if a red or blue shift would occur as the environment of Cy5.5-mannotetraose became less polar, absorbance and emission maxima were recorded after its dissolution in water, TBS, MeOH, and DMSO using a plate reader. To observe how the polarity and salt concentration of the environment affects the fluorescence emission intensity of Cy5.5-mannotetraose, varying solvents and buffers such as MeOH, DMSO, 0.05 M TBS (pH 7.4, with 150 mM sodium chloride), 0.1 M sodium bicarbonate buffer (pH 8.5), and 0.01 M PBS (pH 7.4, with 0.0027 M potassium chloride, and 0.137 M sodium chloride) were added at varying percentages in DI water with a final volume of 5 mL. To prepare the stock solution of Cy5.5-mannotetraose, the mass of the dried sample was found using a Mettler Toledo XPR6U Mass Comparator and dissolved in DI water to yield a concentration of 10 µM. The percent change in fluorescence emission intensity was calculated by comparing the peak emission values for 100 nM Cy5.5-mannotetraose in DI water versus the diluted buffer or solvent solutions. The plate reader settings and experimental design are further described in the Supporting Material.

### 2.5. Optimization of Glucose Sensing

All glucose solutions were prepared in 0.05 M TBS (pH 7.4) and the plate-reader settings and equipment were identical to methods previously described (λex: 655 nm and λem: 698–714 nm).

To identify the best ratio of the Cy5.5-mannose to PEG-ConA, the change in the peak fluorescence emission intensity was monitored by varying the concentrations of PEG-ConA with a constant concentration of each Cy5.5-mannose sample (100 nM) and adding a physiologically high concentration of glucose to each assay sample (800 mg/dL). The assay solutions were prepared in 2 mL volumes and for each sample, 300 μL volumes were injected into 6 wells. The percent change in fluorescence was then calculated using the peak emission values for the wells containing glucose and the wells containing TBS using the experimental methods described in the Supporting Information.

Upon determining the best concentration of PEG-ConA to select for maximum glucose sensitivity with 100 nM of the Cy5.5-mannose, a range of PEG-ConA concentrations were selected to confirm the competitive binding-based glucose response throughout the physiological glucose range (25 to 400 mg/dL). Due to no glucose response being seen with the Cy5.5-mannobiose assay, it was not included in the following studies. By following the procedures outlined in the Supporting Information Section, the percent change in fluorescence intensity was calculated for each glucose concentration and graphed.

### 2.6. Glucose Sensing through Skin

After determining the glucose sensitivity of the assay, its detection within the subcutaneous region was then determined. Tissue samples of rat skin were obtained through the Tissue Share Program at Texas A&M University. These samples were identified as thin (~0.9 mm) and thick (~1.8 mm) and had varying skin pigmentations (light, medium, and dark), roughly equivalent to 1, 4, and 5 on a Fitzpatrick skin tone scale. Each of the samples were excited with a laser diode with a purchased specification of a center wavelength of 690 nm, but measured at 682 nm with a full width at half maximum (FWHM) of 4.5 nm. An Ocean Optics USB4000 Spectrometer was used to collect the emission spectra. For each study, 2.5 µL of the samples were injected into thin plastic hollow tubing of a 10 mm length and 1.3 mm outer diameter and the tubing ends were sealed with black electrical tape to prevent loss of the solutions or contamination to the set-up or tissue samples. The controls for each study were TBS injected into the plastic tubing and sealed. A holder was designed using a black rubber mat to ensure that each sample was placed in the same position and it also allowed for the rat skin to be pinned to keep consistent placement.

Initial testing focused on determining the minimum concentration required to obtain an emission signal of Cy5.5-mannotetraose when placed beneath the rat skin of 2 thicknesses (~0.9 and ~1.8 mm) at a 2.5 µL volume. The concentration range of 20 to 40 µM was seen as ideal for the following studies and the glucose response was confirmed by evaluating the change in fluorescence intensity of various concentration ratios of Cy5.5-mannotetraose and PEG-ConA with 800 mg/dL glucose using the optical benchtop system. Glucose response within the 50 to 400 mg/dL glucose range was then further analyzed for the assay concentration ratios of Cy5.5-mannotetraose and PEG-ConA at 20:15 µM and 40:30 µM and a best fit was found to calculate the percent MARD. The 20:15 µM assay was then chosen to be tested beneath the thinner rat skin sample (~0.9 mm) with glucose concentrations ranging from 100 to 400 mg/dL to ensure the trend holds even with loss of signal intensity caused by the tissue sample.

Skin pigmentation was also a factor of interest and tissue samples that contained darker pigmentation were much thicker due to collection limitations (~2.1 mm) which required a higher concentrated sample of Cy5.5-mannotetraose to produce a detectable signal. Tubing containing the 40:30 µM assay without glucose present was placed beneath tissue samples of similar thickness and the fluorescence intensity at 700 nm was compared to that of tubing containing tbs placed beneath the same skin samples. The percent increase in intensity was analyzed for each skin pigmentation (light, medium, and dark) and an ANOVA test was completed to determine if a significant difference was present.

## 3. Results

### 3.1. Binding Studies of PEG-ConA and Cy5.5-Mannose

**Figure 2:**
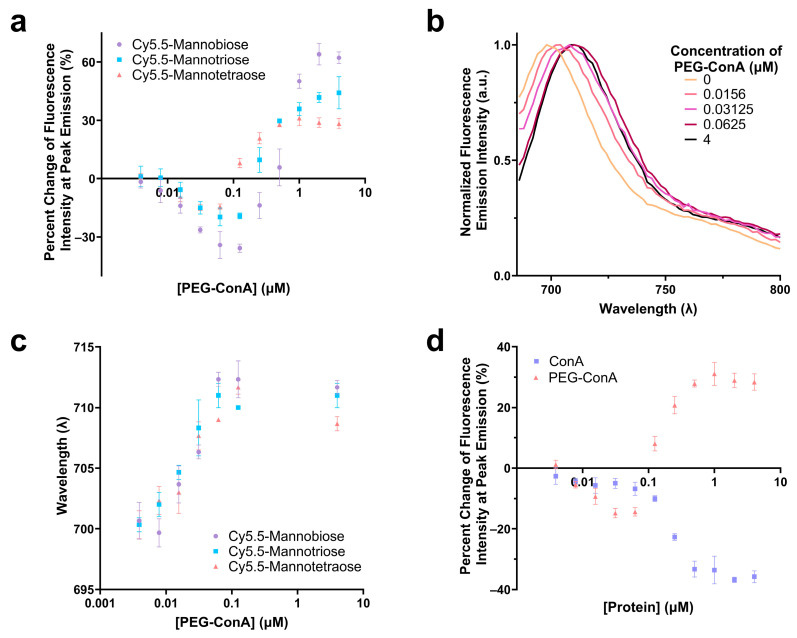
Comparison of the change in fluorescence intensity of Cy5.5-mannotetraose, Cy5.5-mannotriose, and Cy5.5-mannobiose in various concentrations of PEG-ConA (**a**). Emission spectra of Cy5.5-mannotetraose (**b**) and wavelength of peak fluorescence emission intensity for each of the Cy5.5-mannose complexes (**c**) in respect to PEG-ConA concentration. Change in fluorescence intensity of Cy5.5-mannotetraose in PEG-ConA versus ConA at peak emission (**d**). Error bars represent triplicate samples. The error bars represent the standard deviation (*n* = 3).

The performance of the three synthesized Cy5.5-mannose in binding with PEG-ConA was characterized and compared. The percent change in the fluorescence intensities at the peak emissions of Cy5.5-mannotetroase, Cy5.5-mannotriose, and Cy5.5-mannobiose were analyzed as a function of PEG-ConA concentration, and a similar trend was observed for the three dye–sugar conjugates (Figure 2a). As depicted, an increase in the number of mannose molecules from mannobiose to mannotetraose resulted in a decrease in the response sensitivity to PEG-ConA across all PEG-ConA concentrations. A red shift was detected for the emission spectra of Cy5.5-mannotetraose as higher a concentration of PEG-ConA was present (Figure 2b,c) and a similar trend followed for both Cy5.5-mannotriose and Cy5.5-mannobiose (Figure 2c) up to about 0.1 µM and then leveled off for all three. The absorbance and emission maxima were identical to Cy5.5-mannotetraose when dissolved in water versus TBS, showing that by conducting studies in TBS to improve the stability of our PEG-ConA, we are not influencing the absorbance and emission wavelength maxima and instead all wavelength shifts were due to its interactions/binding to PEG-ConA (Table S1). The influence that PEGylation of ConA played on the fluorescence emission intensity of Cy5.5-mannotetraose was determined by comparing it to the response seen with unmodified ConA at identical concentrations (Figure 2d). The absence of PEG chains resulted in quenching only. The different trends of the two types of ConA indicated the PEGylation of ConA altered its surface feature and, thus, the response of the Cy5.5-mannose. This effect was investigated and is discussed in the following section.

### 3.2. Characterization of the Effect of Solvents on the Cy5.5-Mannose

**Figure 3:**
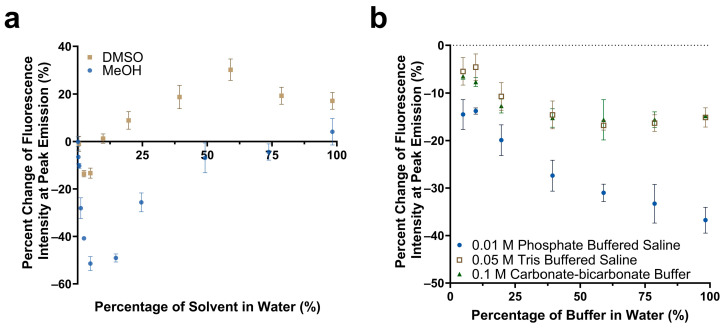
Comparison of the change in fluorescence emission of Cy5.5-mannotetraose at the peak intensity in various concentrations of DMSO versus methanol (**a**). Change in peak fluorescence intensity of Cy5.5-mannotetraose in PBS, TBS, and carbonate-bicarbonate buffers (**b**). The error bars represent the standard deviation (*n* = 3).

The impact of solvent polarity and salt concentration on the fluorescence emission intensity of Cy5.5-mannotetraose was studied. Various dilutions of methanol and DMSO in water were prepared to create an array of environments of varying degrees of polarity. The solvent polarity parameter ET(30) of water, methanol, and DMSO are 63.1, 55.4, and 45.1, respectively, with water being the most polar [39]. For both solvents chosen, a trend was observed of a quenching effect followed by a reversal as the solvent became less polar due to the increased presence of either methanol or DMSO (Figure 3a). Significant reduction of approximately 51% in the fluorescence emission intensity occurred for methanol in comparison to DMSO, and the final emission intensity at 99% methanol was comparable to that of only water. A more noticeable percent increase in the fluorescence emission occurred with DMSO, with a plateau occurring at approximately 19%. The observed trend follows that of the phenomenon of reverse solvatochromism. In lower polarity solvents, positive solvatochromism occurs as the solvents become more polar (moving from right to left in Figure 3a). At some medium polarity points, a reversal is seen and a negative solvatochromism trend follows as the solvents become more polar (again moving from right to left in Figure 3a) [40]. The trends seen for Cy5.5-mannotetraose in dilutions of DMSO are similar to that of increasing concentrations of PEG-ConA. The solvatochromism occurs when Cy5.5-mannose may take advantage of the hydrophilic or hydrophobic cavities present in PEG-ConA upon binding to provide a localized signal [16]. In summary, the synthesized Cy5.5-mannose was responsive to polarity and could generate a change in the fluorescent intensity when it bound/unbound with PEG-ConA.

The response of Cy5.5-mannotetraose in various common biological buffers was also of interest for this system (Figure 3b). Three buffer solutions were diluted with DI water resulting in environments of varying salt concentrations. In all cases, a decrease in the percent change in the fluorescence intensity or a quenching effect was observed as the presence of salt was increased within each of the buffers. For TBS (0.05 M) and carbonate-bicarbonate buffer (0.1 M), the response signal stayed constant within 40%–100% buffer in water. A bigger response signal change in the buffers with 40%–100% of PBS (0.01 M) indicated Cy5.5-mannotetraose was more sensitive to the concentration of phosphate than Tris and carbonate groups. Thus, TBS and carbonate-bicarbonate buffer, particularly if at percentages above 40% in water, are better for the consistent performance of the Cy5.5-mannotetraose. This quenching is likely due to salt-induced aggregation of the cyanine dye caused by the change in the ionic strength of the environment.

### 3.3. Optimization of Glucose Sensing

**Figure 4:**
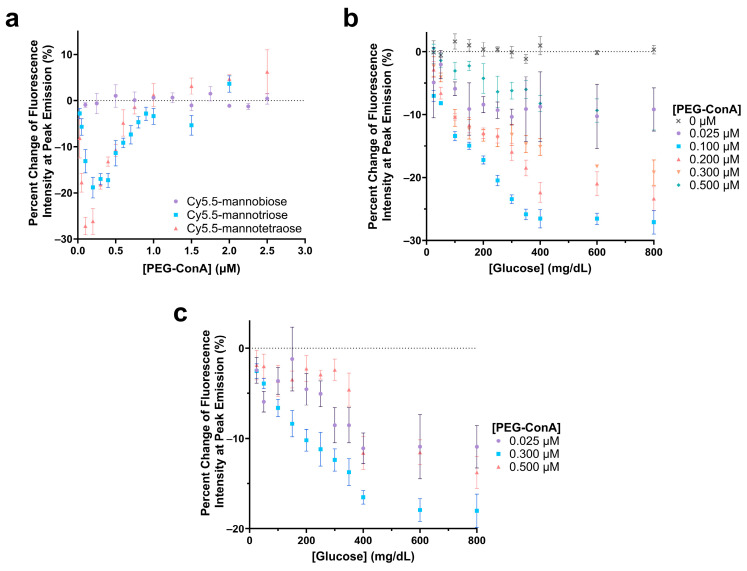
Evaluation of the change in fluorescence intensity at the peak emission due to the addition of 800 mg/dL glucose for 100 nM Cy5.5-mannotetroase, Cy5.5-mannotriose, and Cy5.5-mannobiose with varying concentrations of PEG-ConA (**a**). Glucose response of 0.1 µM Cy5.5-mannotetraose (**b**) and 0.1 µM Cy5.5-mannotriose (**c**) with glucose concentrations ranging 25 to 800 mg/dL and with varying PEG-ConA concentrations. The error bars represent the standard deviation (*n* = 3).

The polarity-sensitive Cy5.5-labeled mannose molecules were then paired with PEG-ConA for glucose sensing. The Cy5.5-labeled mannose molecules functioned as a competing ligand to compete with glucose to bind with PEG-ConA. In competitive binding, the amount of glucose, competing ligands, and the binding sites are important for good sensing performance. The glucose response of the sensor with Cy5.5-mannobiose, Cy5.5-mannotriose, and Cy5.5-mannotetraose was studied with concentrations of PEG-ConA from 0 to 2.5 µM to determine the ratio of Cy5.5-mannose to PEG-ConA in the presence of 800 mg/dL glucose that would yield the most sensitive response (Figure 4a). For this glucose response study, a competitive binding trend is seen where the recognition molecule, PEG-ConA, becomes overly concentrated, and the mannose sugars and glucose no longer need to compete for binding sites within the physiological glucose range. Similar to results found in our previous work [34], Cy5.5-mannobiose yielded no response to glucose, while Cy5.5-mannotriose and Cy5.5-mannotetraose displayed a sensitive response. The high binding affinity of Cy5.5-mannobiose to PEG-ConA in the competitive binding reaction made it difficult for glucose to compete for the binding site, and thus Cy5.5-mannobiose showed virtually no response as a function of PEG-ConA concentration in the presence of 800 mg/dL glucose. The greatest glucose sensitivity was seen for the assay configuration of 100 nM Cy5.5-mannotetraose and a concentration range of 0.1 μM to 0.2 μM PEG-ConA.

The sensitivity of the glucose-sensing assays was further analyzed by observing the responses across a range of PEG-ConA concentrations with a glucose range of 25 to 800 mg/dL for the one that was most sensitive in Figure 4a, namely, Cy5.5-mannotetraose (Figure 4b). Although less sensitive, for completeness, we also ran a limited data set for Cy5.5-mannotriose (Figure 4c). In both cases, a consistent, nearly linear trend was noted where a decrease in fluorescence intensity occurred between 0 and 350 mg/dL before reaching a plateau. This limits the sensing capabilities of the assay to be below 350 mg/dL, and while this still covers the bulk of the concentration range of glucose useful for the majority of people with diabetes, there are individuals with hyperglycemic hyperosmolar syndrome who can regularly have levels exceeding 600 mg/dL [41]. Additionally, the assay compositions with lesser glucose sensitivity consistently resulted in higher errors. To ensure that the glucose solution was not impacting the fluorescence intensity of Cy5.5-mannotetraose, a control study was conducted with 0 µM PEG-ConA, and no change was observed. For 0.1 µM Cy5.5 mannotetraose, the PEG-ConA concentration that yielded the most sensitive glucose response within the 25 to 400 mg/dL glucose range was 0.1 µM, which follows that expected from the response in Figure 4a.

**Figure 5:**
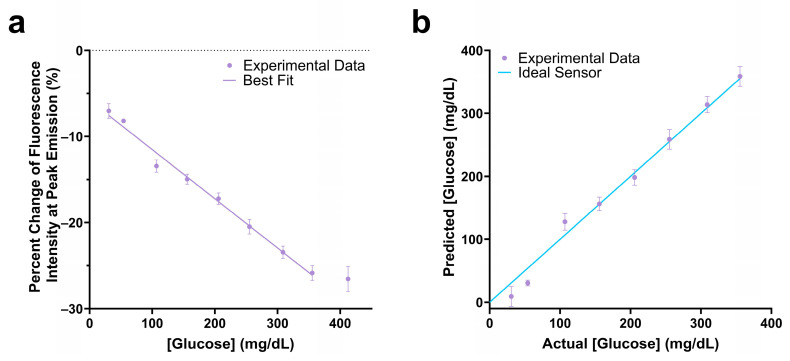
Change in fluorescence intensity at peak emission of the 100 nM Cy5.5-mannotetraose and 100 nM PEG-ConA assay composition within the physiological glucose range (25–400 mg/dL) with a linear fit (**a**). The actual versus predicted glucose concentration of the glucose response shown in (**a**), resulting in a percent MARD of 12.72% in the range 25 to 350 mg/dL (**b**). The error bars represent the standard deviation (*n* = 3).

The assay mixture with 0.1 µM Cy5.5-mannotetraose and 0.1 µM PEG-ConA showed a −25.9% change in fluorescence intensity in response to 350 mg/dL glucose and had a linear response within the 25 to 350 mg/dL glucose range (Figure 5a). The actual glucose concentration was then determined using a YSI 2900 Biochemistry Analyzer and plotted against the predicted concentration (Figure 5b). A standard error of calibration of 11.88 mg/dL and mean absolute relative difference (MARD) of 12.72% was calculated within the glucose range of 25 to 350 mg/dL glucose. The 90% confidence interval values of Figure 5b were calculated and are listed in Table S2.

To assess the effect of a higher concentration of Cy5.5-mannotetraose for the assay, the glucose responses of the assay using two different concentrations of Cy5.5-mannotetraose (0.5 μM and 1 μM) were tested. The results are shown in Figure S1. For 0.5 μM Cy5.5-mannotetraose, the greatest sensitivity was seen when paired with 0.5 μM PEG-ConA, which showed a change in fluorescence intensity emission of −44.9% in the presence of 800 mg/dL glucose. An even better sensitivity was seen for 1 μM Cy5.5-mannotetroase with 1 μM PEG-ConA (−53.5%). These assay configurations were then tested with the physiological range of glucose (25–400 mg/dL) and the sensitivity had improved significantly when compared to the 0.1:0.1 μM assay shown in Figure 4b, but the dynamic range became nonlinear between 250 and 300 mg/dL (Figure S1b).

### 3.4. Glucose Sensing through Skin

**Figure 6:**
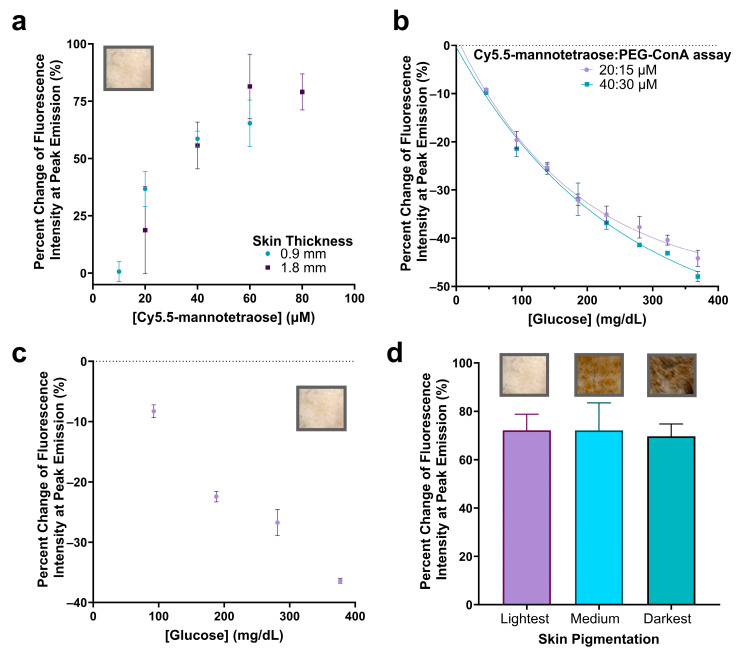
Percent change in fluorescence peak emission of Cy5.5-mannotetraose at varying concentrations when injected into plastic tubing (2.5 μL) and placed beneath the lightest pigmented rat skin tissue of thicknesses ~0.9 and ~1.8 mm (**a**). Confirmation of glucose response at higher assay ratio concentrations of Cy5.5-mannotetraose and PEG-ConA (20:15 μM and 40:30 μM) (**b**). Glucose response of 20:15 μM assay beneath thinner skin sample (~0.9 mm) (**c**). Comparison of percent change in peak emission intensity of 40:30 μM assay without glucose present placed beneath skin samples of the same thickness (~2.1 mm) and of three pigmentations (lightest, medium, and darkest) (**d**). The error bars represent the standard deviation (*n* = 3).

In order to increase the fluorescence emission intensity to ultimately integrate this assay into a subcutaneous implantable biosensor under the skin, we decided to significantly increase the concentration of Cy5.5-mannotetraose and assess if the response with varying PEG-ConA in the presence of 800 mg/dL glucose still holds. A concentration of Cy5.5-mannotetraose required to detect a signal beneath samples of rat skin was determined by dissolving Cy5.5-mannotetraose in TBS which would represent its most quenched state. For the thinner (~0.9 mm) and thicker (~1.8 mm) skin samples, minimal concentrations of 20 and 40 μM were selected, respectively, to conserve the sample while still maintaining an effective brightness (Figure 6a). The 90% confidence interval values of Figure 6a can be found in Table S3.

The assay configurations were again determined by measuring the change in fluorescence emission intensity with varying concentrations of PEG-ConA in the presence of 800 mg/dL glucose but for the higher 20 and 40 μM Cy5.5-mannotetraose (Figure S2). The most sensitive assays for the 20 and 40 μM Cy5.5-mannotetraose were determined to be 15 and 30 μM PEG-ConA with percent changes of −58.8% and −56.4%, respectively, when introduced to 800 mg/dL glucose. The physiological glucose range from 25 to 400 mg/dL was tested and shown to have high sensitivity but also showed a nonlinear trend and fit more to an exponential decay (Figure 6b). The two combinations (20:15 μM and 40:30 μM) showed a similar response to glucose, and their sensitivity was better than the combinations with 0.1–1 μM Cy5.5-mannotetraose. The results confirmed the response to glucose still held after significantly increasing the concentration of the assay components and the greatest percent changes in fluorescence intensity seen within the physiological glucose range was for the 40:30 μM assay at −47.9%. The ConA concentration used here is much lower than the reported level of a ConA-based assay used in subcutaneous skin tissue, which was shown not to pose any health risk [26]. The standard error of calibrations for the 20:15 μM and 40:30 μM assays were calculated to be 12.29 mg/dL and 16.79 mg/dL and percent MARD values were 7.46% and 7.22%, respectively, for a glucose range from 45 to 368 mg/dL. This is an improved percent MARD when compared to the previous lower Cy5.5-mannotetraose to PEG-ConA ratios with the added advantage of a much brighter signal. The 90% confidence interval values for Figure 6b can be found in Table S4.

To confirm the glucose response when placed beneath the rat skin, the thinner skin (~0.9 mm) was first paired with the 20:15 μM assay and tested with several glucose concentrations. The raw spectra data were collected (Figure S3) and the percent change in fluorescence emission intensity was calculated and plotted versus glucose concentration (Figure 6c). The 90% confidence interval values for Figure 6c can be found in Table S5. Additionally, the impact of rat skin pigmentation on the fluorescence intensity was studied by comparing the percent change in the fluorescence emission when the 40:30 μM assay was placed beneath the skin compared to TBS (Figure 6d). The percent change in peak emission intensity was not significantly different (*p* < 0.05) when placed beneath skin samples of different pigmentations. The raw spectra were collected (Figure S4) and a lower baseline measurement was detected for the darkest skin pigmentation most likely due to greater absorption [12]. The 90% confidence interval values for Figure 6d can be found in Table S6. The developed assay was compared to other assays aiming for minimally invasive subcutaneous glucose detection (listed in Table S7) [42,43,44,45,46]. In contrast to others, this work demonstrated successful signal detection of a glucose-sensing probe under skin samples of different pigmentations, which is critical for the reliability of the assay for a range of subjects.

## 4. Conclusions

A far-red fluorescent probe was developed, and it was paired with PEG-ConA to detect the concentration of glucose using competitive binding. The synthesized far-red fluorescent probe, Cy5.5-mannose, was polarity-sensitive and generated fluorescent intensity change when bound/unbound with PEG-ConA. By analyzing the percent change in the fluorescence emission of the three candidates’ (Cy5.5-mannobiose, Cy5.5-mannotriose, and Cy5.5-mannotetraose) reactions to their environment and their competitive binding nature when glucose was introduced, it was determined that Cy5.5-mannotetraose is the most viable option as it yielded the greatest sensitivity to glucose. The response to glucose was detectable through rat skin using a benchtop optical setup, suggesting that the assay has promise as a subcutaneous sensor. In addition, the fluorescent probe and biosensor response is not statistically different for skin pigmentations when comparing the changes in the percent fluorescence intensity, indicating the potential reliability of the probe and the biosensor for subcutaneous glucose monitoring under various types of skin. While significant progress has been made, further research is necessary for integrating the assay into a sensing system for continuous glucose monitoring. Specific future research includes assay cytotoxicity testing, incorporation of the assay into a small biocompatible semipermeable membrane, lifespan study, development of a high sensitivity portable reader, as well as in vivo performance testing.

## Data Availability

The data presented in this study are available upon request.

## Supplementary Materials

The following supporting information [25,42,43,44,45,46] can be downloaded at https://www.mdpi.com/article/10.3390/bios13080788/s1. Table S1: Comparison of absorbance and emission maxima for Cy5.5-mannotetraose in water and TBS; Table S2: Confidence interval values (α = 0.1) for the actual versus predicted glucose concentration of the glucose response of the 100 nM Cy5.5-mannotetraose and 100 nM PEG-ConA assay composition shown in Figure 5b; Figure S1: Determination of the most sensitive ratio of 0.5 µM and 1 µM Cy5.5-mannotetraose and PEG-ConA in response to 800 mg/dL glucose (A). The best ratios were then chosen and tested across the physiological glucose range (B); Table S3: Confidence interval values (α = 0.1) for the percent change in fluorescence intensity at peak emission when varying concentrations of Cy5.5-mannotetraose were placed beneath the lightest rat skin tissue of thicknesses ~0.9 and ~1.8 mm; Figure S2: Determination of most sensitive response to 800 mg/dL glucose for concentration ratio of Cy5.5-mannotetraose and PEG-ConA. Concentrations of Cy5.5-mannotetraose were chosen at 20 and 40 μM and tested with varying concentrations of PEG-ConA; Table S4: Confidence interval values (α = 0.1) for the percent change in fluorescence intensity at peak emission for higher assay ratio concentrations of Cy5.5-mannotetraose and PEG-ConA when tested at glucose concentration ranges within the physiological range; Table S5: Confidence interval values (α = 0.1) for the percent change in fluorescence intensity at peak emission of the 20:15 µM Cy5.5-mannotetraose:PEG-ConA assay in response to glucose within the physiological range; Figure S3: Fluorescence emission spectra of the 20:15 μM Cy5.5-mannotetraose:PEG-ConA assay demonstrating the response to glucose beneath thinner rat skin; Figure S4: Fluorescence emission spectra comparing the 40:30 μM Cy5.5-mannotetraose:PEG-ConA assay to a TBS control when placed beneath skin samples of three different pigmentations (lightest, medium, and darkest) with a thickness of ~2.1 mm; Table S6: Confidence interval values (α = 0.1) for the percent change in fluorescence intensity at peak emission of the 40:30 µM Cy5.5-mannotetraose:PEG-ConA assay without glucose present when placed beneath thicker skin samples of different pigmentations; Table S7: Comparison of different glucose sensors aiming for minimally invasive subcutaneous glucose detection.
