# Supplementary material for: A Polarity-Sensitive Far-Red Fluorescent Probe for Glucose Sensing through Skin
Source: Biosensors (Basel). 2023 Aug 4;13(8):788. doi: 10.3390/bios13080788 (PMC10452146; doi:10.3390/bios13080788)
Supplement: Supplementary file 1 [file biosensors-13-00788-s001.zip › biosensors-2503696-supplementary.pdf]

biosensors

Supplementary Material

A Polarity-Sensitive Far-Red Fluorescent Probe for Glucose Sensing through Skin

Lydia Colvin [1,*], Dandan Tu [1], Darin Dunlap [1], Alberto Rios [1] and Gerard Coté [1,2]

[1] Department of Biomedical Engineering, Texas A&M University, College Station, TX 77843, USA
[2] Center for Remote Health Technologies and Systems, Texas A&M Engineering Experiment Station, College Station, TX 77843, USA
* Correspondence: ledcolvin@tamu.edu

Synthesis of Cy5.5-mannose and PEG-ConA

The Cy5.5 labeled mannose molecules were synthesized using a linker molecule and a three-step procedure. Firstly, the mannose molecules were individually added to the lowest volume of DMSO:Acetic Acid (7:3 v/v) required for dissolution of 1 molar equivalent mannose, 10 molar equivalents of the linker, 1-(N-Boc-aminomethyl)-4-(aminomethyl)benzene, and 10 molar equivalents of NaBH3CN. After 5 days reacting in an oil bath set at 36°C, the samples were dried via lyophilization, high-performance liquid chromatography (HPLC) purified, and the binding of the mannose molecules to the linker was confirmed by positive electrospray ionization mass spectrometry (+ESI MS). Next, the BOC protective group on the linker molecule was removed by dissolving the dried samples in TFA, TIPS, and water (95%, 2.5%, 2.5% by volume) and shaken vigorously for 2 h. The product was precipitated by cold diethyl ether and centrifuged, collected, and dried before confirming via (+)ESI. Lastly, with the BOC protective group now removed, a primary amine was available for binding to Cy5.5 NHS ester. The reaction occurred in DMF for 24 h and consisted of 1 molar equivalent of mannose-linker, 2 molar equivalents of Cy5.5 NHS ester, and 4 molar equivalents of DIPEA. It was HPLC purified and the product was confirmed via (+)ESI. The final concentration of each Cy5.5-mannose conjugate was determined by measuring the peak absorbance at approximately 680 nm with a Cary 300 UV-Visible spectrophotometer from Agilent Technologies (California, US) and using the extinction coefficient of 198,000 M-1 cm-1.

PEGylation of ConA was conducted through utilization of the primary amines on ConA. Upon complete dissolution of ConA in 0.1 M pH of 8.5 sodium bicarbonate buffer (10 mg mL-1), MaM was added to protect the binding sites and mixed slowly for 20 min. The mPEG-SPA was added at 32 molar equivalents per 1 molar equivalent of ConA and mixed slowly for 4 h on a rocking platform shaker and then removed and allowed to continue reacting for at least 24 more hours at room temperature. The sample was washed 7 times with Amicon Ultra-2 Centrifugal Filters (30 kDa MWCO, 2 mL) with TBS (pH 7.6, 50 mM Tris-HCl and 150 mM NaCl) at 3,000 x g for 20 min at 4°C using an Eppendorf Centrifuge 5810R. PEGylation was confirmed via matrix-assisted laser desorption ionization time-of-flight mass spectrometry (MALDI-TOF MS) and potential aggregation was monitored via dynamic light scattering (DLS). The final concentration of PEG-ConA was determined by measuring the peak absorbance at 280 nm with a Cary 300 UV-Vis spectrophotometer and using the extinction coefficient of 118,560 M-1 cm-1 [1].

Binding Studies of PEG-ConA and Cy5.5-mannose

The 11 serial dilutions of PEG-ConA were made using TBS beginning with 24 μM of PEG-ConA with a final volume of 1 mL. Within minutes prior to each run, 250 μL of Cy5.5-mannotetraose (120 nM) was injected into 6 wells with 50 μL of PEG-ConA added to 3 alternating wells and 50 μL of TBS added to the remaining wells to serve as the control. This would yield a final concentration of 100 nM Cy5.5-mannotetraose for each well. Each

well was then mixed thoroughly before beginning the fluorescence emission scan and the process was repeated for each PEG-ConA concentration and again with Cy5.5-mannotriose and Cy5.5-mannobiose.

Characterization of the Effect of Solvents on the Cy5.5-mannose

For comparing the fluorescence intensity of Cy5.5-mannotetraose in varying environments, 297 μL of the buffer or solvent mixtures were added to 3 alternating wells and 297 μL of DI water were added to the remaining wells. Then, 3 μL of 10 μM Cy5.5-mannotetraose was added to each well, yielding a final volume of 300 μL with a final concentration of 100 nM Cy5.5-mannotetraose. The plate reader was then used to conduct a fluorescence intensity scan (λex: 655 nm and λem: 698 - 714 nm). The peak emission intensities were then determined and the percent change in the fluorescence intensity was calculated by comparing the initial condition of Cy5.5-mannotetraose in DI water to that of the final condition, varying percentages of the buffer or solvent.

Optimization of Glucose Sensing

For determining the best ratio of Cy5.5-mannose to PEG-ConA, in alternating wells, 10 μL of TBS or glucose was added to have a final concentration of glucose equal to 800 mg/dL and minimal dilution of the assay. Each well was then thoroughly mixed before the fluorescence emission scan was conducted. This experiment was repeated using identical methods with 0.5 μM and 1 μM Cy5.5-mannotetraose to identify the best concentration of Cy5.5-mannose needed in the assay.

For measuring the glucose response among the physiological glucose range, solutions of 100 nM Cy5.5 mannotetraose and 0, 25, 100, 200, 300, and 500 nM PEG-ConA were made at a 35 mL volume and for each glucose concentration measurement, 300 μL of the assay was added to 6 wells and in alternating wells, either 10 μL of TBS or 10 μL of the glucose solutions were added. After mixing thoroughly, a fluorescence intensity emission scan was taken with the previously described settings and the percent change in the fluorescence intensity was calculated. This method was then repeated for 100 nM Cy5.5-mannotriose with 25, 300, and 500 nM PEG-ConA, 0.5 μM Cy5.5-mannotetraose with 0.5 μM PEG-ConA, and 1 μM Cy5.5-mannotetraose with 1 μM PEG-ConA using the same glucose stock solutions. To better analyze its glucose response, the glucose concentrations were verified via a YSI 2900 Biochemistry Analyzer. A linear fit was found for the plot and used to predict the glucose concentrations.

Absorbance and Emission of Cy5.5-mannotetraose in water versus TBS

Table S1. Comparison of absorbance and emission maxima for Cy5.5-mannotetraose in water and TBS.

Solvent	λ_{abs} (nm)	λ_{em} (nm)
Water	677	699
TBS	677	699

Statistical analysis of glucose response of 100:100 nM Cy5.5-mannotetraose:PEG-ConA assay

Table S2. Confidence interval values (α = 0.1) for the actual versus predicted glucose concentration of the glucose response of the 100 nM Cy5.5-mannotetraose and 100 nM PEG-ConA assay composition shown in Figure 5b.

Actual [Glucose] (mg/dL)	Predicted [Glucose] (mg/dL)	
	Mean	90% CI [Lower limit, Upper limit]
25	9.2	[-17.7, 36.1]
50	30.7	[22.9, 38.4]
100	127.9	[105.4, 150.4]
150	156.2	[138.0, 174.4]
200	198.2	[177.7, 218.8]
250	258.7	[232.4, 285.1]
300	313.9	[291.9, 335.8]
350	358.8	[332.2, 385.3]

The effect of the concentration of Cy5.5-mannotetraose

To determine the effect of an increasing concentration of Cy5.5-mannotetraose for the assay, the glucose responses of the assay using different concentrations of Cy5.5-mannotetraose (0.5 µM and 1µM) were tested using the plate reader. The results are shown in Figure S1. The greatest response was determined for 500 nM and 1 µM of Cy5.5 mannotetraose with varying concentrations of PEG-ConA in the presence of glucose at 800 mg/dL (Figure S1a). It was concluded that the two best combinations of Cy5.5-mannotetraose:PEG-ConA were 0.5:0.5 µM and 1:1 µM. These combinations were then tested with the physiological glucose range of 25-400 mg/dL (Figure S1b).

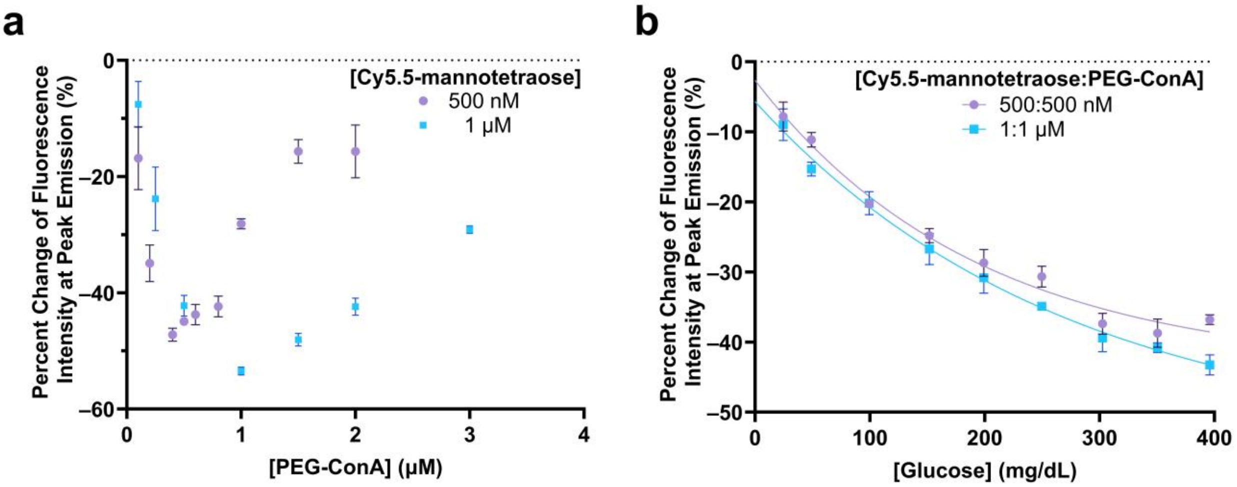

Figure S1. Determination of the most sensitive ratio of 0.5 µM and 1 µM Cy5.5-mannotetraose and PEG-ConA in response to 800 mg/dL glucose (a). The best ratios were then chosen and tested across the physiological glucose range (b).

Statistical analysis of Cy5.5-mannotetraose concentration required for detectable signal through thinner and thicker rat skin samples

Table S3. Confidence interval values (α = 0.1) for the percent change in fluorescence intensity at peak emission when varying concentrations of Cy5.5-mannotetraose were placed beneath the lightest rat skin tissue of thicknesses ~0.9 and ~1.8 mm.

[Cy5.5-mannotetraose] (μM)	Percent Change in Fluorescence Intensity at Peak Emission (%)			
	0.9 mm Skin Thickness		1.8 mm Skin Thickness	
	Mean	90% CI [Lower limit, Upper limit]	Mean	90% CI [Lower limit, Upper limit]
10	0.7	[-6.7, 8.0]	--	--
20	36.7	[23.9, 49.5]	18.8	[-13.4, 50.9]
40	58.6	[53.0, 64.1]	55.7	[38.4, 72.9]
60	65.4	[48.3, 82.4]	81.4	[57.7, 105.2]
80	--	--	79.0	[65.7, 92.3]

Assay evaluation at higher concentrations using the optical benchtop system

The concentration of Cy5.5-mannotetraose was increased to 20 μM and 40 μM and was combined with PEG-ConA with concentrations ranging from 5 to 60 μM. The percent change in fluorescence emission of these combinations in the presence of 800 mg/dL glucose was calculated (Figure S2). The assay was excited using the laser diode, and the emission was detected using the spectrometer. For 20 μM and 40 μM, the PEG-ConA concentrations that led to the greatest glucose sensitivity were 15 μM and 30 μM, respectively.

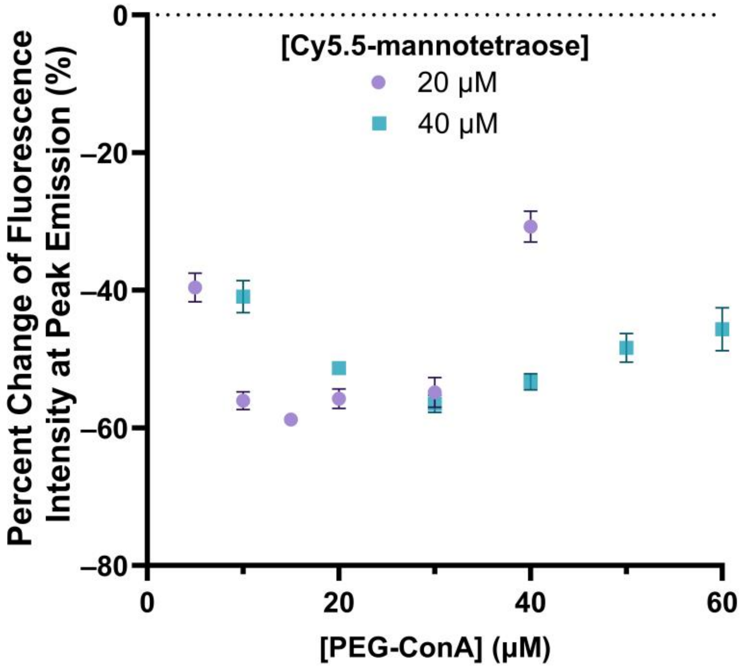

Figure S2. Determination of most sensitive response to 800 mg/dL glucose for concentration ratio of Cy5.5-mannotetraose and PEG-ConA. Concentrations of Cy5.5- mannotetraose were chosen at 20 and 40 μM and tested with varying concentrations of PEG-ConA.

Statistical analysis of glucose response at higher assay ratio concentrations within a physiological glucose concentration range

Table S4. Confidence interval values (α = 0.1) for the percent change in fluorescence intensity at peak emission for higher assay ratio concentrations of Cy5.5-mannotetraose and PEG-ConA when tested at glucose concentration ranges within the physiological range.

[Glucose] (mg/dL)	Percent Change in Fluorescence Intensity at Peak Emission (%)			
	20:15 μM Cy5.5-mannotetraose:PEG-ConA assay		40:30 μM Cy5.5-mannotetraose:PEG-ConA assay	
	Mean	90% CI [Lower limit, Upper limit]	Mean	90% CI [Lower limit, Upper limit]
50	-9.2	[-9.4, -8.9]	-9.6	[-10.7, -8.4]
100	-19.6	[-22.6, -16.6]	-21.4	[-24.2, -18.6]
150	-25.3	[-26.6, -24.1]	-25.5	[-27.6, -23.4]
200	-32.0	[-33.9, -30.1]	-31.9	[-37.6, -26.2]
250	-35.1	[-38.0, -32.1]	-36.8	[-39.1, -34.5]
300	-37.7	[-41.5, -33.9]	-41.4	[-41.8, -41.0]
350	-40.4	[-42.1, -38.7]	-43.1	[-43.3, -42.9]
400	-44.2	[-47.0, -41.4]	-47.9	[-49.6, -46.3]

Statistical analysis of glucose response of assay beneath thinner rat skin

Table S5. Confidence interval values (α = 0.1) for the percent change in fluorescence intensity at peak emission of the 20:15 μM Cy5.5-mannotetraose:PEG-ConA assay in response to glucose within the physiological range.

[Glucose] (mg/dL)	Percent Change in Fluorescence Intensity at Peak Emission (%)	
	Glucose Response of 20:15 μM assay (Cy5.5-mannotetraose:PEG-ConA) beneath thinner rat skin (~0.9 mm)	
	Mean	90% CI [Lower limit, Upper limit]
25	-10.3	[-12.6, -8.1]
50	-27.9	[-29.8, -26.0]
100	-33.2	[-37.8, -28.7]
150	-45.3	[-46.1, -44.4]

Fluorescence emission spectra of the assay with glucose beneath rat skin

The assay containing 20 μM Cy5.5-mannotetraose, 15 μM PEG-ConA, and varying concentrations of glucose were pipetted into plastic tubing and placed under the thinner rat skin (~0.9 mm). Using the laser diode, the emission spectra was obtained for each concentration of glucose using the benchtop system (Figure S3).

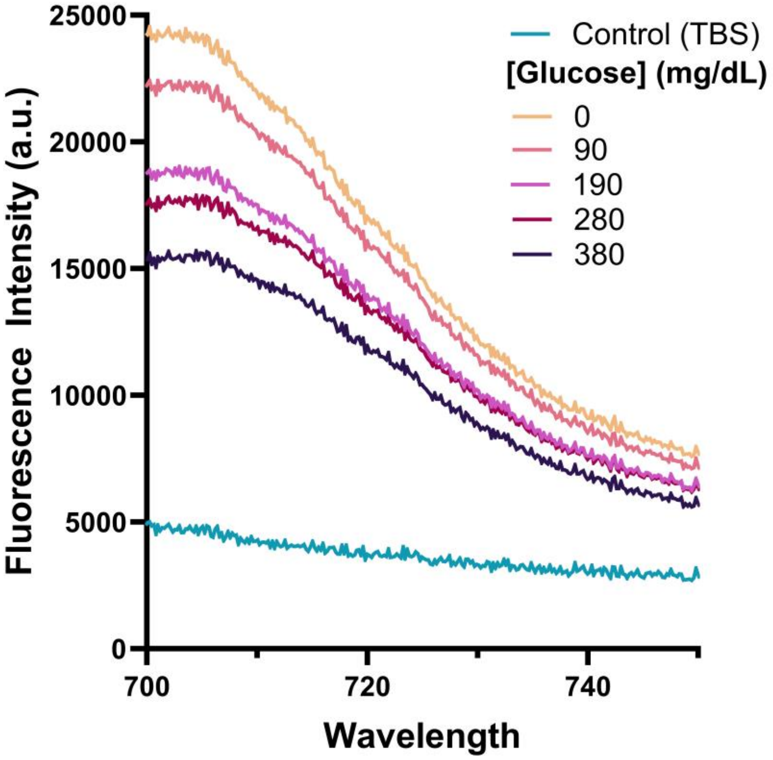

Figure S3. Fluorescence emission spectra of the 20:15 μM Cy5.5-mannotetraose:PEG-ConA assay demonstrating the response to glucose beneath thinner rat skin.

Fluorescence emission spectra under skin with different pigmentations

The assay containing 40 μM Cy5.5-mannotetraose and 30 μM PEG-ConA was placed into plastic tubing under thicker rat skin with varying pigmentations to examine the impact of skin pigmentations on the emission intensity (Figure S4). There was almost no difference between the lightest skin tone and the medium skin tone; however, there was a decrease in the fluorescence intensity when placed under the darkest skin tone, most likely due to greater absorption.

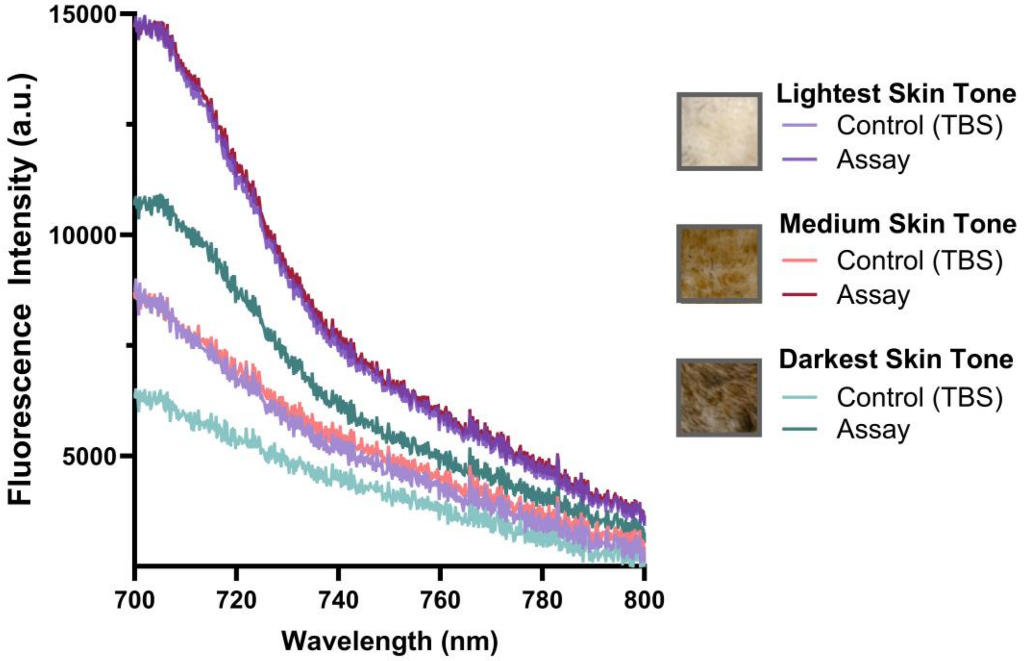

Figure S4. Fluorescence emission spectra comparing the 40:30 μM Cy5.5-mannotetraose:PEG-ConA assay to a TBS control when placed beneath skin samples of three different pigmentations (lightest, medium, and darkest) with a thickness of ~2.1 mm.

Statistical analysis of comparison of assay emission intensity under skin with different pigmentations

Table S6. Confidence interval values (α = 0.1) for the percent change in fluorescence intensity at peak emission of the 40:30 μM Cy5.5-mannotetraose:PEG-ConA assay without glucose present when placed beneath thicker skin samples of different pigmentations.

	Percent Change in Fluorescence Intensity at Peak Emission (%)	
**Skin Pigmentation**	**Mean**	**90% CI [Lower limit, Upper limit]**
Lightest	72.1	[61.0, 83.2]
Medium	72.1	[53.0, 91.3]
Darkest	69.6	[60.9, 78.3]

Comparision of different glucose sensors

Table S7. Comparison of different glucose sensors aiming for minimally invasive subcutaneous glucose detection.

References	Year	Probe Design	Detection Range	Accuracy	Performance for different skin pigmentation
This work	2023	Polarity-sensitive far-red fluorescent probe based on Cy5.5-mannotetraose and PEG-ConA	25 – 350 mg/dL	MARD of 12.72%	Probe signal detection not significantly different when tested with skin samples of different pigmentations.
[2]	2022	Polyacrylamide hydrogel matrix embedded with luminescent polymer dots (Pdots)	2 – 20 mM (36 – 360 mg/dL)	Not available.	Not available.
[3]	2020	Silica nanoparticles with IRDye680 and a boronic acid–viologen boronic acid embedded in poly(2-hydroxyethyl methacrylate) scaffolds	50 – 600 mg/dL	Not available.	Not available.
[4]	2022	Enzyme-driven phosphorescence lifetime-based glucose-sensing assay embedded in a thermoresponsive membrane	50 – 200 mg/dL	Not available.	Not available.
[5]	2020	Surfaced enhanced Raman scattering (SERS) nanosensor based on gold nanoparticles functionalized with 4-mercaptophenylboronic acid (MPBA)	2– 20 mM (36 – 360 mg/dL)	Not available.	Not available.
Eversense E3 [6]	2022	Fluorophores and microfluorometer encapsulated in a porous membrane	40-400 mg/dL	MARD of 8.5%	No effect

References

1. Locke, A.K.; Cummins, B.M.; Abraham, A.A.; Cote, G.L. PEGylation of concanavalin A to improve its stability for an in vivo glucose sensing assay. *Analytical chemistry* **2014**, *86*, 9091-9097.
2. Liu, J.; Fang, X.; Zhang, Z.; Liu, Z.; Liu, J.; Sun, K.; Yuan, Z.; Yu, J.; Chiu, D.T.; Wu, C. Long-term in vivo glucose monitoring by polymer-dot transducer in an injectable hydrogel implant. *Analytical Chemistry* **2022**, *94*, 2195-2203.

3. Le, L.V.; Chendke, G.S.; Gamsey, S.; Wisniewski, N.; Desai, T.A. Near-Infrared Optical Nanosensors for Continuous Detection of Glucose. *Journal of Diabetes Science and Technology* **2020**, *14*, 204-211, doi:10.1177/1932296819886928.
4. Dong, P.; Ko, B.S.; Lomeli, K.A.; Clark, E.C.; McShane, M.J.; Grunlan, M.A. A glucose biosensor based on phosphorescence lifetime sensing and a thermoresponsive membrane. *Macromolecular Rapid Communications* **2022**, *43*, 2100902.
5. Huang, Y.; Luo, Y.; Liu, H.; Lu, X.; Zhao, J.; Lei, Y. A subcutaneously injected SERS nanosensor enabled long-term in vivo glucose tracking. *Engineered Science* **2020**, *14*, 59-68.
6. Garg, S.K.; Liljenquist, D.; Bode, B.; Christiansen, M.P.; Bailey, T.S.; Brazg, R.L.; Denham, D.S.; Chang, A.R.; Akturk, H.K.; Dehennis, A.; et al. Evaluation of Accuracy and Safety of the Next-Generation Up to 180-Day Long-Term Implantable Eversense Continuous Glucose Monitoring System: The PROMISE Study. *Diabetes Technol Ther* **2022**, *24*, 84-92, doi:10.1089/dia.2021.0182.
